# Transcriptome based Identification of silver stress responsive sRNAs from Bacillus cereus ATCC14579

**DOI:** 10.6026/97320630015474

**Published:** 2019-07-31

**Authors:** Jayavel Sridhar, Manickam Gayathri

**Affiliations:** 1 Department of Biotechnology (DDE), Madurai Kamaraj University, Madurai-625021, Tamil Nadu, India

**Keywords:** sRNA, Transcriptome, Bacillus cereus ATCC 14579, Silver stress

## Abstract

Microbes modulate their metabolic and physiological mechanisms in response to changing environmental conditions. It is our interest to identify small regulatory RNAs using microarray expression data (GSE26043) obtained from B. cereus ATCC 14579 in AgNO3 stress. By definition, expression of transcripts from the Intergenic Regions (IGR) with >=2 fold under silver stress is predicted as novel small RNAs. Computational analysis of the IGR expression levels extracted from the available microarray data help in the identification of stress responsive sRNAs with rare promoters (Sigma 24, 28, 32, 54 and 70) followed by terminator signals predicted using the sRNAscanner tool. We predicted 1512 sRNA specific regions on both positive and negative strands collectively. Thus, a non-redundant high scoring unique 860 sRNAs with distinct promoter (S24: 83, S28: 86, S32: 31, S54: 57, S70: 223, sRNA_specific_S70: 380) and terminator signals are reported. These unique computationally predicted sRNA regions were verified with the highly expressing IGRs from the microarray data. It should be noted that 14 sRNAs reported in earlier studies were also found in this dataset. This study has reported 71 additional sRNAs from the transcriptome under metal stress response. Hence, we use global transcriptomics data for the identification of novel sRNAs in B. cereus. We described a general model using a procedure for the identification of small regulatory RNAs using microarray expression data with appropriate cross validation modules. It is found that some sRNAs reported in this study were found to have multiple rare promoters. This opens the possibility of sRNA activation under multiple stress condition. These sRNA data reported in this study should be characterized for their mRNA targets and molecular functional networks in future investigations.

## Background

Microbes are often exposed to the changing environmental conditions such as low temperature, oxidative stress and heavy metal stress, etc [1-6]. To adapt in such drastic environments, it modulates the physiological and metabolic networks. During evolution they have developed various adaptive mechanisms to maintain the cellular integrity [2]. These mechanisms allow microbes to survive and function in new, unfavorable conditions. These molecular mechanisms developed within the bacteria against different stress conditions are termed as stress response [1]. The main objective of stress response is to protect the cellular components from various stress conditions that leads to damage of DNA, RNA and protein. Stress response may be exhibited through changes in the metabolic activities by producing specific regulatory molecules to activate or suppress the synthesis of particular protein to maintain the physiological conditions. The result of these changes will show as temporary slowdown or stoppage of the cell division, morphological changes, etc.

One such drastic environmental condition is heavy metal ions exposure like Ag+, Cd2+, Hg2+, Co2+, Cu2+, Ni2+, Zn2+ that causes toxic effects in the microbial cell [4]. To reduce the toxic nature of metal ions, bacteria accumulate the ions into nano particles [7]. The exact mechanism for the synthesis of nanoparticles employing biological agents has not been revealed yet. This is because of various biological agents involves in the synthesis of the nano particles. There are two approaches to synthesis the nano particles using microbes. First one is intracellular synthesis, where the nano particles synthesized by the microbes accumulates within the cell after the transportation of metal ions. Another mode is extracellular synthesis, using the extracellular enzymes secreted by the microbes. For example, silver nano particles are synthesized by the B.cereus by bioreduction process. The extracellular reductase enzyme helps in the reduction of silver ion into nano form [8],[9].

Small untranslated regulatory RNAs are identified in all forms of life. In eukaryotes it is referred as noncoding RNAs (ncRNAs) and prokaryotic counterparts are called as sRNAs [10]. Recently the role of sRNAs was detected in several important metabolic and physiological functions in microbes such as regulation of sporulation[11],sugar metabolism [12][13], iron homeostasis [14],survival under oxidative stress [15], DNA damage repair,maintenance of cell surface components [16] and regulation of pathogenicity [17]. Thus, sRNAs play important regulatory role in gene expression in prokaryotes. Sizes of the sRNAs were ranges from 50 to 400 nucleotides.

The chromosomally encoded sRNA regulate the mRNA targets in response to the drastic environmental conditions like low temperature, heavy metal exposure, iron homeostasis, quorum sensing, outer membrane protein modification, etc. They function as either positive or negative regulators of the gene expression. Several bioinformatics and experimental approaches have been developed for genome wide identification of sRNAs. Information on sRNAs was reported in BSRD database [18]; sRNAmap [19] and sRNAdb [20]. So far, the regulatory sRNAs can be classified into two types based on its function. The first class of sRNAs is alternate the protein activity by binding to the translational regulatory proteins like CsrB, 6S, and GlmY. Another class of sRNA regulates the expression of mRNA by direct base-pairing with its target such as GcvB and RyhB. The mRNA binding sRNAs can be either cis (highly complementarity) or trans (partial complementarity) mode.

Transcriptional factors are the proteins bind at a particular site in the upstream region of the gene, involve in the initiation of transcription by enabling the binding of RNA polymerase. Sigma factors are the exclusive regulators for transcription initiation in microbes. Based on the sequence analysis, major sigma factors are classified into sigma 70 (S70), sigma 54 (S54) and extra cytoplasmic function (ECF) sigma factors [21-24] such as S24, S28, S32, S38, S19 etc. Each sigma factor plays specific role in particular physiological and environmental conditions. For example S70 involves in the regulation of housekeeping genes expression and S24 regulates genes involved in extreme heat stress management. Also these ECF sigma factors are expected to initiate sRNA gene expression from IGRs under various environmental conditions. Genomic microarray is the global hybridization technique to study the expression of entire transcriptome in real time. It has also been used to quantify the expression levels of sRNA/mRNA in terms of transcript copy numbers. We are attempting to identify silver stress responsive sRNAs by analyzing the publicly available transcriptomic microarray data from NCBI-GEO [25] of B.cereus.

## Methodology

### Retrieval and analysis of Microarray expression data:

The global expression profile [GSE26043] of the control and AgNO3 exposed test samples at various time intervals were collected from NCBI-GEO database. The expression data has 15000 oligo probes to monitor protein coding (10450) and Intergenic regions (4550) in both sense and anti-sense directions. Details about the targeting gene/mRNA coordinates and respective probe information were collected from the complete genome of B. cereus ATCC14579 (NC_004722). We have created specific PERL script to calculate the coordinates for empty IGRs from the above protein coding table. The normalized global expression pattern of coding and IGRs were also collected from the above Affymetrix data. We have selected the IGR regions showing expression values >=2 fold compared with the control sample and proposing these regions to express intergenic transcripts.

### Creating training PWMs for rare promoters:

Binding nucleotides along with their specific spacer regions of various sigma factors were collected [26] and used as training data set to predict the stress regulating sRNAs. Positional Weight Matrices (PWMs) reflecting the consensus binding sequence for different promoters (Sigma 24, 28, 32, 54 and 70) were generated for each promoter with the available C module (PWM_create.cpp) in sRNAscanner package.

### Stress responsive sRNA detection using sRNAscanner:

**Figure 1:**
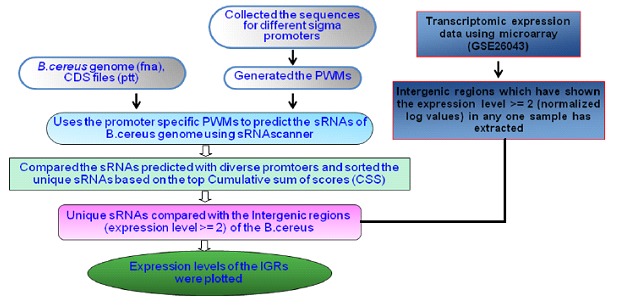
Methodology used for the identification of sRNAs in stress response

Complete genome sequence of the B. cereus ATCC 14579 (NC_004722.fna) and corresponding coding region file (NC_004722.ptt) were retrieved from GenBank [27]. Rhoindependent terminator dataset was used along with any one of the stress responsive promoter data to predict the intergenic sRNA regions using sRNAscanner tool. Predictions of sRNAs were carried out in both sense and antisense directions of the genome. The screening was done in the intergenic regions and the sRNA length for prediction was set to 40-350 nucleotides. Rest of the parameters were given as the default values, i.e. The cut-off value is 2 for -35 and -10 box matrices, for terminator.txt.matrix is 3; spacer range between [-35] and [-10] promoter boxes: 12-18; unique hit value: 200; minimum cumulative sum of score (CSS): 14. All the computational predictions were carried out in Ubuntu workstation with 3.2 GHz speed and 8 GB RAM. Overall methodology used in the identification of stress responsive sRNAs is illustrated in Figure 1.

### Verification of the predicted sRNA regions with target IGRs

**Figure 2:**
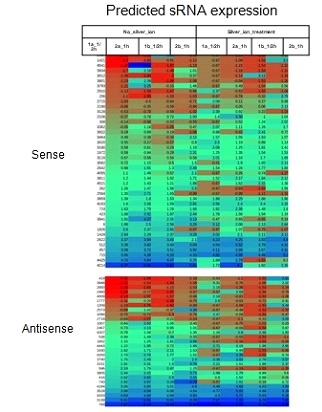
Expression levels of the predicted sRNAs at various time points were taken from the microarray data and presented in the form of a heat maps

We have overlapped the predicted sRNA locations with the intergenic region coordinates retrieved from the genome microarray by using the In-house developed awk script. If, any part of sRNA matches with the intergenic coordinates showing expression in the microarray were confirmed as sRNA encoding IGR. Expression levels of these sRNA harboring IGRs at various time points were taken from the microarray data and presented in the form of a heat maps (Figure 2).

## Results and Discussion:

Bacterial sRNAs are novel regulators of gene expression involved in diverse biological processes. A change in the environment often causes physiological stress and bacteria cope with that stress by altering the expression of relevant genes and producing new proteins, which may allow the cell to repair damage or protect itself in future. This stress-induced gene expression response is often mediated by proteins called sigma factors [28]. Recent report identified the multiple sigma factor regulated sRNAs in Agrobacterium spp. [29] which provoke us to study the silver stress responsive sRNAs in B. cereus. Before predicting the sRNAs based on the transcription site it is necessary to identify different sigma factors that are being encoded by respective genomes [29].

This study is aiming to identify novel stress responsive sRNAs using virtual comparison of computational sRNA predictions with global transcriptome data collected under silver stress condition. The genome wide microarray experiment in B. cereus ATCC 14579 was designed with custom designed oligo probes against CDS's and IGR's to monitor their expression under various time intervals [30]. It was reported that, the expression of IGR's were highly down regulated (20%) at 30 and 60 mins.

**Figure 3:**
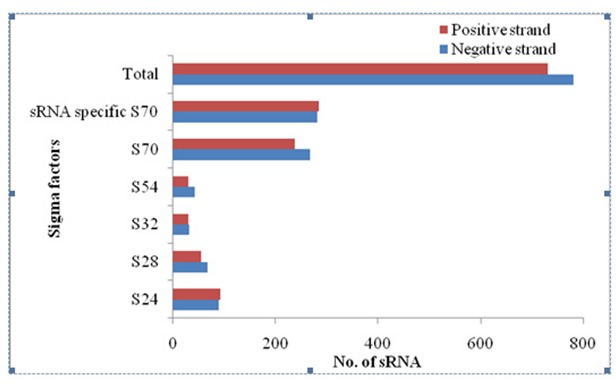
Genome wide prediction of sRNA with rare sigma factors in positive and negative strands of Bacillus cereus using sRNAscanner tool.

We have constructed the promoter datasets for S24, S28, S32, S54 and S70 using the PWM_create module given in sRNAscanner suite. We have applied these PWMs with their corresponding spacer regions in sRNAscanner to predict the intergenic sRNAs under predefined parameters [31]. Above search has detected 1512 sRNAs spanned under sigma factors ie. S24, S28, S32, S54, S70 in Bacillus cereus genome. Among these sRNAs, 781 were transcribed from negative strand and 731 from positive strand (Table 1 and Figure 3). Overlapping sRNAs were further analyzed based on their CSS values and non-redundant unique sRNAs were identified (Table 2).

Most of the sRNAs were found to be regulated at high frequency by sRNA specific σ 70 (567) followed by σ 70 (506) and σ 24 (183) than σ 28, σ 32 and σ 54. Initially 567 sRNAs were predicted to be transcribed by sRNA specific σ70, among them 380 sRNAs were found to be unique and it may play a major role in stress tolerance. Expression profiles of the 367 IGRs within the microarray data have shown significant transcripts (Table 3). Among them 42 regions in sense IGRs and 29 regions in antisense IGRs were computationally predicted as sRNAs.

**Figure 4:**
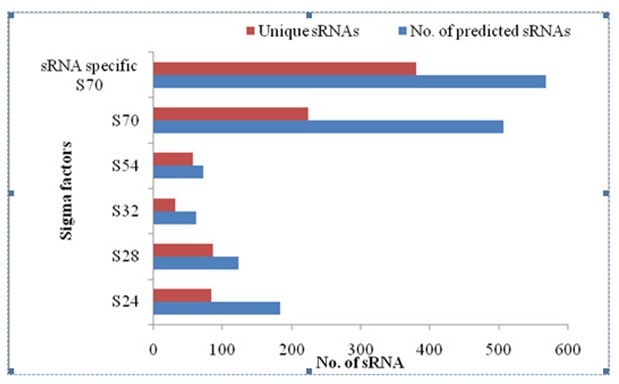
Distribution of redundant and unique sRNAs predicted to have different promoters

**Figure 5:**
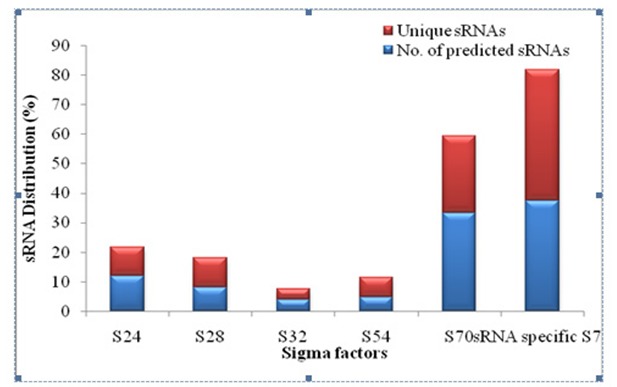
Overall distribution of the predicted and unique sRNAs

The total number of unique sRNAs in predicted sequences for each sigma factor of Bacillus spp. was shown in Figure 4.sRNA specific S70 and S70 exhibit 380 and 223 sRNAs followed by other sigma factors. The sRNA distribution in both predicted and unique sequences implies that sRNA specific S70 showed 44.19 % of unique sRNAs followed by S70 (25.93%) and other sigma factors (Figure 5).A computer-based search identified 23 SR1 homologues in several bacterial genera including Bacillus subtulis. All homologues share a high structural identity with Bacillus subtilis SR1 [32]. Bacterial cells harbor a variety of non-coding RNAs depend on the type of stress response. Therefore, it is necessary to validate the predicted sRNAs with their targets as to whether they are really silver stress responsive sRNAs.

## Conclusion

The present study clearly demonstrated the use of global transcriptomics data for the identification of novel sRNAs in B. cereus. This methodology may be applied in any model organism supported with the microarray and genome data. Proposed methodology also retained fourteen novel sRNAs reported in earlier studies, which clearly validated the reliability of the applied method. Interestingly, few sRNAs reported in this study were found to have multiple rare promoters and it opens the possibility of sRNA activation under multiple stress condition. Remaining, sRNAs reported in this study may be functionally characterized for their mRNA targets and molecular functional networks.

## Figures and Tables

**Table 1:** Total number of predicted sRNAs with diverse promoters

S24	S28	S32	S54	S70	sRNA specific S70	Total
No. of Predicted sRNA	183	123	61	72	506	567	1512
Negative strand	90	68	31	42	268	282	781
Positive strand	93	55	30	30	238	285	731

**Table 2:** Unique sRNAs identified with different promoters

S24	S28	S32	S54	S70	sRNA specificS70	Total
Negative strand	34	44	13	32	123	184	430
Positive strand	49	42	18	25	100	196	430
Total	83	86	31	57	223	380	860

**Table 3:** Number of IGRs matching with the predicted sRNAs

Expression level >=2	367	Predicted	Unpredicted
Sense IGR	280	42*	238
Antisense IGR	87	29*	58
